# Factors Associated With Cognitive Improvement After Bariatric Surgery Among Patients With Severe Obesity in the Netherlands

**DOI:** 10.1001/jamanetworkopen.2023.15936

**Published:** 2023-05-30

**Authors:** Debby Vreeken, Florine Seidel, Emma M. Custers, Lisette Olsthoorn, Sophie Cools, Edo O. Aarts, Robert Kleemann, Roy P. C. Kessels, Maximilian Wiesmann, Eric J. Hazebroek, Amanda J. Kiliaan

**Affiliations:** 1 Department of Medical Imaging, Anatomy, and Radboud Alzheimer Center, Radboud University Medical Center, Donders Institute for Brain, Cognition, and Behavior, Center for Medical Neuroscience, Nijmegen, the Netherlands; 2 Department of Bariatric Surgery, Vitalys, Rijnstate Hospital, Arnhem, the Netherlands; 3 Department of Metabolic Health Research, Netherlands Organisation for Applied Scientific Research, Leiden, the Netherlands; 4 Department of Surgery, WeightWorks Clinics, Amersfoort, the Netherlands; 5 Department of Medical Psychology and Radboud Alzheimer Center, Radboud University Medical Center, Donders Institute for Brain, Cognition, and Behavior, Nijmegen, the Netherlands; 6 Department of Neuropsychology and Rehabilitation Psychology, Centre for Cognition, Radboud University, Donders Institute for Brain, Cognition, and Behavior, Nijmegen, the Netherlands; 7 Vincent van Gogh Institute for Psychiatry, Venray, the Netherlands; 8 Division of Human Nutrition and Health, Wageningen University, Wageningen, the Netherlands

## Abstract

**Importance:**

Bariatric surgery–induced weight loss is often associated with improved cognitive function. However, improvement in cognitive function is not always exhibited by all patients, and the mechanisms behind cognitive improvement remain unknown.

**Objective:**

To investigate the association of changes in adipokines, inflammatory factors, mood, and physical activity with alterations in cognitive function after bariatric surgery among patients with severe obesity.

**Design, Setting, and Participants:**

This cohort study included 156 patients with severe obesity (body mass index [calculated as weight in kilograms divided by height in meters squared], >35) eligible for Roux-en-Y gastric bypass, aged between 35 and 55 years, who were enrolled in the BARICO (Bariatric Surgery Rijnstate and Radboudumc Neuroimaging and Cognition in Obesity) study between September 1, 2018, and December 31, 2020. Follow-up was completed July 31, 2021; 146 participants completed the 6-month follow-up and were included in the analysis.

**Intervention:**

Roux-en-Y gastric bypass.

**Main Outcomes and Measures:**

Overall cognitive performance (based on a 20% change index of the compound *z* score), inflammatory factors (eg, C-reactive protein and interleukin 6 levels), adipokines (eg, leptin and adiponectin levels), mood (assessed via the Beck Depression Inventory), and physical activity (assessed with the Baecke questionnaire).

**Results:**

A total of 146 patients (mean [SD] age, 46.1 [5.7] years; 124 women [84.9%]) completed the 6-month follow-up and were included. After bariatric surgery, all plasma levels of inflammatory markers, including C-reactive protein (median change, −0.32 mg/dL [IQR, –0.57 to –0.16 mg/dL]; *P* < .001) and leptin (median change, −51.5 pg/mL [IQR, –68.0 to –38.4 pg/mL]; *P* < .001), were lower, whereas adiponectin levels were higher (median change, 0.15 μg/mL [IQR, –0.20 to 0.62 µg/mL]; *P* < .001), depressive symptoms were (partly) resolved (median change in Beck Depression Inventory score, −3 [IQR, –6 to 0]; *P* < .001), and physical activity level was higher (mean [SD] change in Baecke score, 0.7 [1.1]; *P* < .001). Cognitive improvement was observed in 43.8% (57 of 130) of the participants overall. This group had lower C-reactive protein (0.11 vs 0.24 mg/dL; *P* = .04) and leptin levels (11.8 vs 14.5 pg/mL; *P* = .04) and fewer depressive symptoms at 6 months (4 vs 5; *P* = .045) compared with the group of participants who did not show cognitive improvement.

**Conclusions and Relevance:**

This study suggests that lower C-reactive protein and leptin levels, as well as fewer depressive symptoms, might partly explain the mechanisms behind cognitive improvement after bariatric surgery.

## Key Points

**Question:**

Are changes in adipokines, inflammatory factors, mood, and physical activity associated with cognitive improvement 6 months after bariatric surgery?

**Findings:**

In this cohort study of 156 participants with severe obesity who underwent bariatric surgery, 43.8% of the participants experienced overall cognitive improvement 6 months after surgery. This group had lower C-reactive protein and leptin levels and fewer depressive symptoms 6 months after surgery compared with the group of participants who did not show cognitive improvement.

**Meaning:**

This study suggests that reduced C-reactive protein and leptin levels, as well as fewer depressive symptoms, might partly explain the mechanisms behind cognitive improvement after bariatric surgery.

## Teaser

This cohort study investigates the association of changes in adipokines, inflammatory factors, mood, and physical activity with alterations in cognitive function after bariatric surgery.

## Introduction

Obesity is a worldwide major health challenge.^1^ It is associated with metabolic disorders, and obesity in midlife is considered a risk factor for cognitive decline and dementia at later ages.^2,3^

Obesity seems to affect multiple cognitive domains, such as memory, verbal fluency, and executive functions.^4^ Weight loss after bariatric surgery is associated with improved cognition.^5,6^ Improvements in memory and executive functions have been found 3 months to 3 years after bariatric surgery.^7,8^ However, not all studies of bariatric surgery reported cognitive improvement,^9,10^ and the mechanisms behind such improvement are not well established yet, to our knowledge. Some proposed mechanisms are changes in white adipose tissue and related adipokines, reduced systemic inflammation, and improved mood and physical activity.^5,11,12,13^ Classification of patients into those with improved cognition and those without cognitive improvement might reveal more information on the potential underlying mechanisms.

White adipose tissue is an endocrine organ regulating secretion of adipokines and cytokines, such as leptin, adiponectin, and proinflammatory cytokines.^14^ In obesity, the secretion of proinflammatory adipokines is increased, resulting in systemic low-grade inflammation.^15,16^ These adipokines and proinflammatory cytokines may influence structural and functional changes in the brain.^15^ However, associations between other inflammatory factors, such as C-reactive protein (CRP) level, and cognitive function among people with obesity are inconsistent. An association between higher levels of CRP and impairment in cognitive flexibility was observed,^17^ or it was found for women only,^18^ while other studies failed to replicate this finding.^19^ Leptin has been shown to be an important factor associated with attentional performance among individuals with obesity.^20^ After bariatric surgery, reduced inflammation and changes in adipokines are observed, indicating that reduced inflammation may improve cognition after bariatric surgery. Furthermore, mood and physical activity play a major role in cognitive performance.^12,13^ After surgery, changes in mood and increased physical activity are often observed; therefore, these lifestyle factors may be a mechanistic link between bariatric surgery and cognitive improvement.^5^

In this study, we investigated the association between changes in adipokines, inflammatory factors, mood, and physical activity with cognitive function. Data were collected before and 6 months after bariatric surgery for adults with severe obesity (body mass index [BMI; calculated as weight in kilograms divided by height in meters squared], >35) enrolled in the BARICO (Bariatric Surgery Rijnstate and Radboudumc Neuroimaging and Cognition in Obesity) study. We also compared individuals with improved cognition with those without cognitive improvement. The results provide a better understanding of the potential mechanisms underlying cognitive improvement after bariatric surgery and may help to develop personalized strategies complementary to bariatric surgery.

## Methods

### Study Population

Data from the BARICO study were analyzed.^21^ Between September 1, 2018, and December 31, 2020, patients were recruited at the Rijnstate Hospital (Arnhem, the Netherlands). Participants were aged 35 to 55 years at recruitment and eligible for Roux-en-Y gastric bypass surgery. Neurologic or severe psychiatric illness, pregnancy, and treatment with any antibiotics, probiotics, or prebiotics 3 months before or at any point during the study (excluding preoperative prophylaxis) were exclusion criteria. Follow-up was completed July 31, 2021. Cognitive performance was assessed before and 6 months after surgery using neuropsychological tests. At both time points, blood samples were collected, anthropometric data were recorded, and participants filled out questionnaires. The study was approved by the Medical Ethics Committee CMO region Arnhem–Nijmegen and the local institutional ethics committee. The study was conducted according to the Declaration of Helsinki^22^ and according to the ICH Harmonised Tripartite Guideline for Good Clinical Practice.^23^ All participants provided written informed consent. The study was prospectively registered in the Dutch Trial Registry.^24^ The Strengthening the Reporting of Observational Studies in Epidemiology (STROBE) reporting guideline was followed.

### Anthropometric Data and Blood Samples

Anthropometric measurements included blood pressure, body weight, BMI, waist circumference (WC), and percentage total body weight loss, defined as weight loss divided by total weight before Roux-en-Y gastric bypass surgery. Fasting (≥3 hours) blood samples were collected at both time points. Plasma levels of CRP, leptin, adiponectin, serum amyloid A (SAA), and plasminogen activator inhibitor 1 (PAI-1) were determined with human enzyme-linked immunosorbent assays. Tumor necrosis factor (TNF), interleukin 1β (IL-1β), and interleukin 6 (IL-6) levels were determined with single molecule array technology using the SP-X Imaging and Analysis system (Quanterix).

### Cognitive Outcomes

Cognitive performance was assessed by trained researchers using neuropsychological tests, described elsewhere.^21^ All tests are standardized, demonstrate good validity, and have good retest reliability. In short, general cognition was assessed by the Montreal Cognitive Assessment.^25^ Summed scores of the 3 trials (forward, backward, and sorting) of the Digit Span test (Wechsler Adult Intelligence Scale–Fourth Edition) were used to determine working memory.^26^ Episodic memory was assessed via the immediate and delayed Story Recall Test from the Rivermead Behavioral Memory test.^27^ The Controlled Oral Word Association Test (COWAT) was used to determine verbal fluency.^28^ Last, the Flexibility subtest from the Tests of Attentional Performance, version 2.3, was used to study ability to shift attention.^29^ Two different versions of the Montreal Cognitive Assessment, Story Recall Test, and COWAT for each time point were used to overcome material-specific practice effects. A compound score for global cognitive function was calculated by taking the mean of the *z* scores of the total score of the Digit Span Test, the Story Recall Test, and COWAT and the Flexibility subtest score from the Tests of Attentional Performance. The *z* scores at 6 months were based on the mean (SD) of each test score in the sample at baseline.

To examine individual associations of the surgery with cognitive test outcomes, we calculated the 20% change index.^30^ This index assumes significant cognitive improvement if the postoperative test score is 20% higher than the preoperative test score. The following formula was used to calculate this index: 5(*x*_2_ − *x*_1_)/*x*_1_, where *x*_2_ is the postoperative test score and *x*_1_ the preoperative test score. This score was calculated for each domain separately and for the compound *z* score. An index of 1.00 or more indicates significant improvement.

### Educational Level

Educational level was determined via the Verhage score, using 7 categories (1 = less than primary school; 7 = academic degree) based on the Dutch educational system that are comparable with the International Standard Classification of Education.^31,32^ A score of 4 or less is defined as a low educational level, a score of 5 is defined as a middle educational level, and a score of 6 or 7 is defined as a high educational level.

### Questionnaires

Participants filled in standardized, online questionnaires at both time points. To assess depressive symptoms, the Beck Depression Inventory (BDI) (edition 1a) was used.^33^ The BDI is a 21-item self-reported questionnaire determining the presence of depressive symptoms over the past 2 weeks. Each response is scored on a scale ranging from 0 (absence of symptom) to 3 (severe symptom). A total score from 0 to 9 indicates minimal depression, 10 to 20 indicates mild depression, 21 to 30 indicates moderate depression, and 31 or more indicates severe depression. To assess physical activity, the Baecke questionnaire was used,^34^ which contains 16 questions with a 5-point Likert scale on the time spent on various activities. Three index scores for work, sport, and leisure were combined into 1 total score. The total score ranges from 3 to 15; higher scores indicate a higher physical activity level.

### Statistical Analysis

Statistical analyses were performed using IBM SPSS statistics, version 25 (SPSS Inc). Normality was checked for continuous variables. When assumptions on normality were not met, a natural log transformation or nonparametric test was used. To test changes over time, repeated-measures analyses of variance (ANOVA), the Wilcoxon signed rank test, or the χ^2^ test were used for continuous parametric, continuous nonparametric, and categorical data, respectively. To compare changes in cognitive test scores over time, the following covariates were selected: age, sex, educational level, and preoperative BMI. None of the covariates had a significant association with the change in cognitive test scores or showed interaction with cognitive test scores over time. Therefore, no covariates were added. Statistical tests were 2-sided, and the α level was set at .05.

Next, we examined differences between participants showing overall cognitive improvement compared with those not showing cognitive improvement, based on the 20% change index for the compound *z* score. A participant was classified as improved when his or her index score was 1.00 or more. First, we tested whether the groups differed based on age, sex, and educational level (eTable 1 in Supplement 1). Since this was not the case, we did not add covariates in the model. Repeated-measures ANOVA was performed to test differences between groups regarding weight loss, circulating plasma markers, mood, and physical activity levels with time as within-participant variables and assigned groups as a between-participant variable. Furthermore, we explored the group × time interaction within the model. For nonnormally distributed data, the Mann-Whitney test at 6 months was used. Last, we explored whether changes in cognition were associated with changes in weight loss, circulating plasma markers, mood, and physical activity levels using bivariate correlations.

## Results

### Descriptive Statistics

A total of 146 patients (mean [SD] age, 46.1 [5.7] years; 124 women [84.9%]) were included in the analyses (eFigure 1 and eTable 2 in Supplement 1). Participants’ characteristics are listed in Table 1.^31^ Mean weight, BMI, WC, and blood pressure were significantly lower after surgery. Participants also used significantly less medication for comorbidities, such as diabetes and hypertension, and fewer participants reported use of antidepressants.

**Table 1.** Characteristics of Participants (N = 146)

Characteristic	Baseline	At 6 mo	*F* or χ^2^ value^a^	*P* value
Age, mean (SD), y	46.1 (5.7)	NA	NA	NA
Sex, women, No. (%)	124 (84.9)	NA	NA	NA
Weight, mean (SD), kg	122.2 (16.0)	89.0 (12.9)	2864.65^b^	<.001^b^
BMI, median (IQR)	41.2 (36.7-44.0)	30.0 (27.9-32.8)	3095.64^b^	<.001^b^
TBWL, mean (SD), %	NA	27.2 (4.9)	NA	NA
WC, median (IQR), cm^c^	123 (116-134.8)	98 (91.5-111)	839.69^b^	<.001^b^
Educational level, No. (%)^d^				
Low	14 (9.6)	NA	NA	NA
Middle	79 (54.1)	NA	NA	NA
High	53 (36.3)	NA	NA	NA
Use of medication, No. (%)				
Oral antidiabetics	14 (9.6)	8 (5.5)	79.80^b^	<.001^b^
Insulin therapy	8 (5.5)	3 (2.1)	52.84^b^	<.001^b^
Blood pressure–lowering agents	52 (35.6)	33 (22.6)	69.99^b^	<.001^b^
Lipid-lowering agents	20 (13.7)	13 (8.9)	89.25^b^	<.001^b^
Antidepressants	16 (11.0)	11 (7.5)	96.66^b^	<.001^b^
Blood pressure, median (IQR), mm Hg^e^				
Systolic	136.5 (125-147)	125.0 (114-136)	43.80^b^	<.001^b^
Diastolic	84.0 (80-91)	80.0 (73-85)	24.00^b^	<.001^b^
MoCA score, median (IQR)	27 (24-27)	27 (25-28)	1.83	.18

Abbreviations: BMI, body mass index (calculated as weight in kilograms divided by height in meters squared); MoCA, Montreal Cognitive Assessment; NA, not applicable; TBWL, total body weight loss; WC, waist circumference.

^a^ Repeated-measures analyses of variance or χ^2^ tests were conducted to examine changes in characteristics over time.

^b^ Significant change over time.

^c^ Complete data on both time points were available for 108 participants.

^d^ A Verhage score of 4 or less is defined as low educational level, a Verhage score of 5 as a middle educational level, and a Verhage score of 6 or 7 as a high educational level.^31^

^e^ Complete data on both time points were available for 115 participants.

### Changes in Adipokines and Inflammatory Markers

**Figure 1.**
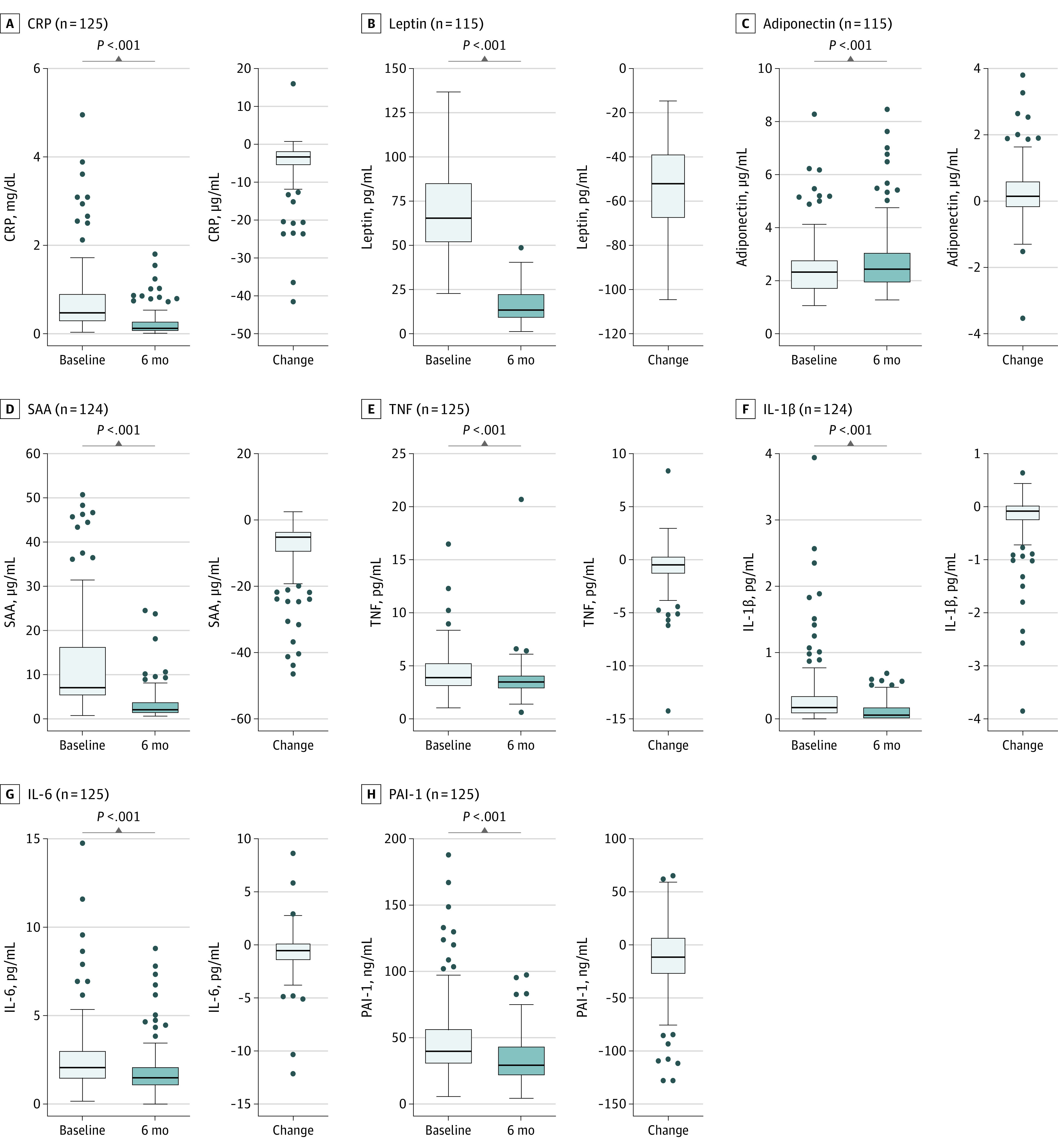
Box Plots With Plasma Concentrations of Adipokines and Inflammatory Markers Before and 6 Months After Bariatric Surgery and the Change Between These 2 Time Points For illustrative purposes, 2 extreme high serum amyloid A (SAA) values (111.49 and 151.18 μg/mL) and 1 interleukin 1β (IL-1β) value (7.59 pg/mL) at the baseline measurement are not shown in the graphs. Repeated-measures analyses of variance were conducted to examine changes of time. The difference over time for IL-1β is based on the Wilcoxon signed rank test. The horizontal bar inside the boxes indicates the median, and the lower and upper ends of the boxes are the first and third quartiles. The whiskers indicate values within 1.5× the IQR from the upper or lower quartile (or the minium and maximum if within 1.5× the IQR of the quartiles), and data more extreme than the whiskers are plotted individually as outliers (circles). CRP indicates C-reactive protein; IL-6, interleukin 6; PAI-1, plasminogen activator inhibitor 1; and TNF, tumor necrosis factor. SI conversion factor: To convert CRP to mg/L, multiply by 10.0.

Changes in plasma adipokine levels and inflammatory markers are depicted in Figure 1. Less inflammation was observed after surgery (eTable 3 in Supplement 1). Furthermore, levels of leptin (median change, −51.5 pg/mL [IQR, –68.0 to –38.4 pg/mL]; *P* < .001), CRP (median change, −0.32 mg/dL [IQR, –0.57 to –0.16 mg/dL] [to convert to mg/L, multiply by 10.0]; *P* < .001), and PAI-1 (median change, –11.2 ng/mL [IQR, –28.0 to 7.6 ng/mL]) were lower, whereas adiponectin levels were higher (median change, 0.15 μg/mL [IQR, –0.20 to 0.62 µg/mL]; *P* < .001).

### Changes in Cognitive Function, Mood, and Physical Activity

Cognitive test scores were higher 6 months after surgery for all cognitive domains (Table 2; eFigure 2 in Supplement 1). Based on the 20% change index, 39.0% of the participants (57 of 146) showed improvement in episodic memory (Story Recall), and 42.3% of the participants (55 of 130) showed an ability to shift attention (Tests of Attentional Performance Flexibility index score). Only 11.0% of the participants (11 of 146) showed improvement in working memory (Digit Span), and 28.1% of the participants (41 of 146) showed improvement in verbal fluency (COWAT). Lower BDI scores were observed (median change, −3 [IQR, –6 to 0]; *P* < .001), indicating improved mood. At baseline, 27.5% of the participants (39 of 142) showed mild depressive symptoms, and 14.1% participants (20 of 142) showed moderate depressive symptoms. Only 8.0% (11 of 138) and 5.8% (8 of 138) of the participants continued to show symptoms of, respectively, mild and moderate depression after surgery. Furthermore, a higher Baecke score was observed after surgery (mean [SD] change, 0.7 [1.1]; *P* < .001), indicating higher physical activity levels.

**Table 2.** Changes in Cognitive Outcomes, Depression Symptoms, and Physical Activity Among Patients Undergoing Bariatric Surgery (N = 146)

Test	Mean (SD) value		*F* or *z* value^a^	*P* value^a^	η^2^ Value^a^	Improved, based on 20% change index, No. (%)
	Baseline	At 6 mo				
Cognition						
Digit Span (sum of forward, backward and sorting)	25.88 (4.82)	26.47 (4.49)	5.39^b^	.02^b^	0.04	16 (11.0)
Story Recall (sum of immediate and delayed recall)	16.83 (6.57)	18.51 (6.42)	10.45^b^	.002^b^	0.07	57 (39.0)
COWAT	37.62 (10.67)	40.86 (11.61)	22.72^b^	<.001^b^	0.14	41 (28.1)
TAP Flexibility index score^c^	−2.85 (8.24)	0.86 (8.44)	35.36^b^	<.001^b^	0.22	55 (42.3)
Compound *z*-score^c^	0.01 (0.68)	0.28 (0.65)	56.55^b^	<.001^b^	0.31	57 (43.8)
BDI score, median (IQR)^d^	9 (5 to 13)	5 (3 to 7)	−6.67^b^	<.001^b^	NA	NA
Minimal, No./total No. (%)	79/142 (55.6)	119/138 (86.2)	NA	NA	NA	NA
Mild, No./total No. (%)	39/142 (27.5)	11/138 (8.0)	NA	NA	NA	NA
Moderate, No./total No. (%)	20/142 (14.1)	8/138 (5.8)	NA	NA	NA	NA
Severe, No. (%)	4/142 (2.8)	0	NA	NA	NA	NA
Baecke score^e^	7.75 (1.21)	8.45 (1.21)	48.77^b^	<.001^b^	0.30	NA

Abbreviations: BDI, Beck Depression Inventory; COWAT, Controlled Oral Word Association Test; NA, not applicable; TAP, Tests of Attentional Performance.

^a^ Repeated-measures analyses of variance or the Wilcoxon signed rank test were conducted to examine changes in characteristics over time.

^b^ Significant change over time.

^c^ Complete data on both time points were available for 130 participants.

^d^ Wilcoxon signed rank test was conducted to examine changes of time. Complete data on both time points were available for 138 participants.

^e^ Complete data on both time points were available for 114 participants.

Next, we divided the group into improvers and nonimprovers based on their overall cognitive improvement, defined as a 20% change index of 1.00 or more for their compound *z* score. In total, 43.8% of the participants (57 of 130) were classified as cognitive improvers who had lower cognitive test scores at baseline compared with nonimprovers (eTable 1 in Supplement 1). After surgery, both groups showed lower BMI, WC, CRP level, leptin level, SAA level, IL-1β level, PAI-1 level, and BDI score, as well as a higher Baecke score (Table 3). Typical of improvers was a more pronounced reduction of median CRP and leptin levels compared with nonimprovers (CRP: improvers, from 0.42 mg/dL [IQR, 0.24-0.70 mg/dL] to 0.11 mg/dL [IQR, 0.05-0.23 mg/dL]; *P* < .001; nonimprovers, from 0.70 mg/dL [IQR, 0.41-1.22 mg/dL] to 0.24 mg/dL [IQR, 0.07-0.41 mg/dL]; *P* < .001; and leptin: improvers, from 63.7 pg/mL [IQR, 47.9-79.5 pg/mL] to 11.8 pg/mL [IQR, 8.0-19.8 pg/mL]; *P* < .001; nonimprovers, from 72.6 pg/mL [IQR, 58.9-90.6 pg/mL] to 14.5 pg/mL [IQR, 9.8-23.2 pg/mL]; *P* < .001). Improvers also showed a lower median BDI score after surgery compared with nonimprovers (improvers, from 8 [IQR, 4-11] to 4 [IQR, 3-6]; *P* < .001; nonimprovers, from 8 [IQR, 5-14] to 5 [IQR, 3-8]; *P* = .045).

**Table 3.** Differences in Anthropometric Measures, Plasma Levels, Mood, and Physical Activity Between Cognitive Improvers and Nonimprovers 6 Months After Bariatric Surgery

Outcome	Within groups^a^								Between groups, repeated-measures ANOVA^a^	
	Improvers (n = 57)				Nonimprovers (n = 73)				*F* or *z* value	*P* value
	Baseline, median (IQR)	At 6 mo, median (IQR)	*F* or *z* value	*P* value	Baseline, median (IQR)	At 6 mo, median (IQR)	*F* or *z* value	*P* value		
Anthropometric measurements										
TBWL, mean (SD), %^b^	NA	27.1 (5.5)	NA	NA	NA	27.1 (4.6)	NA	NA	0.00	.98
BMI	40.9 (38.3 to 43.9)	29.6 (27.1 to 32.7)	973.51^c^	<.001^c^	41.3 (39.8 to 44.1)	30.5 (28.1 to 33.0)	1824.90^c^	<.001^c^	1.73	.19
WC, cm^d^	123.0 (115 to 134)	100.0 (92 to 112)	307.87^c^	<.001^c^	125.0 (116.0 to 136.5)	98.0 (91.0 to 107.8 to)	537.94^c^	<.001^c^	0.03	.87
Blood pressure, mm Hg^e^										
Systolic	137 (127.5 to 147)	120.5 (112 to 135)	5.55^c^	.04^c^	136 (124 to 150)	124 (115 to 138)	19.26^c^	<.001^c^	0.62	.43
Diastolic	84 (80 to 92)	79 (71 to 89)	14.40^c^	<.001^c^	84 (79 to 92)	80 (74 to 86)	8.69^c^	.005^c^	0.43	.51
Plasma levels										
CRP, mg/dL^f^	0.42 (0.24 to 0.70)	0.11 (0.05 to 0.23)	183.17^c^	<.001^c^	0.70 (0.41 to 1.22)	0.24 (0.07 to 0.41)	178.89^c^	<.001^c^	4.48^c^	.04^c^
Leptin, pg/mL^g^	63.7 (47.9 to 79.5)	11.8 (8.0 to 19.8)	458.26^c^	<.001^c^	72.6 (58.9 to 90.6)	14.5 (9.8 to 23.2)	658.48^c^	<.001^c^	4.32^c^	.04^c^
Adiponectin, μg/mL^g^^,^^h^	2.3 (1.7 to 2.9)	2.6 (1.9 to 3.7)	21.19^c^	<.001^c^	2.4 (1.9 to 2.7)	2.4 (2.1 to 2.9)	0.79	.38	NA	NA
SAA, μg/ml^f^	7.0 (5.0 to 11.7)	2.2 (1.1 to 3.7)	153.18^c^	<.001^c^	9.0 (5.8 to 21.6)	2.3 (1.2 to 5.7)	246.70^c^	<.001^c^	2.62	.11
TNF, pg/mL^f^^,^^i^	3.9 (3.0 to 5.2)	3.8 (3.3 to 4.5)	0.76	.39	4.4 (3.4 to 5.7)	3.2 (2.7 to 4.2)	15.65^c^	<.001^c^	NA	NA
IL-1β, pg/mL^h^^,^^j^	0.16 (0.05 to 0.36)	0.04 (0.00 to 0.16)	−3.42^c^	<.001^c^	0.18 (0.08 to 0.38)	0.06 (0.00 to 0.20)	−3.42^c^	<.001^c^	−0.19	.85
IL-6, pg/mL^f^^,^^i^	1.7 (1.3 to 2.8)	1.6 (1.2 to 2.4)	2.09	.16	2.5 (1.4 to 3.6)	1.6 (0.9 to 2.1)	27.6^c^	<.001^c^	NA	NA
PAI-1, ng/mL^f^	43.4 (31.8 to 55.8)	28.5 (23.3 to 44.6)	6.10^c^	.02^c^	38.89 (29.7 to 57.6)	29.1 (19.5 to 42.6)	11.06^c^	.002^c^	0.66	.42
BDI score^h^^,^^k^	8 (4 to 11)	4 (3 to 6)	−4.39^c^	<.001^c^	8 (5 to 14)	5 (3 to 8)	−4.36^c^	<.001^c^	−2.01^c^	.045^c^
Baecke score, mean (SD)^l^	7.7 (1.3)	8.4 (1.3)	17.75^c^	<.001^c^	7.8 (1.3)	8.4 (1.1)	24.63^c^	<.001^c^	0.03	.88

Abbreviations: ANOVA, analysis of variance; BDI, Beck Depression Inventory; BMI, body mass index (calculated as weight in kilograms divided by height in meters squared); CRP, C-reactive protein; IL-1β, interleukin 1β; IL-6, interleukin 6; NA, not applicable; PAI-1, plasminogen activator inhibitor 1; SAA, serum amyloid A; TBWL, total body weight loss; TNF, tumor necrosis factor; WC, waist circumference.

SI conversion factor: To convert CRP to mg/L, multiply by 10.0.

^a^ Repeated-measures ANOVA was conducted to examine differences within and between improvers and nonimprovers.

^b^ *F* value based on a 1-way ANOVA between improvers and nonimprovers.

^c^ Significant change within and between groups.

^d^ Complete data on both time points were available for 98 participants.

^e^ Complete data on both time points were available for 101 participants.

^f^ Complete data on both time points were available for 110 participants.

^g^ Complete data on both time points were available for 100 participants.

^h^ The *z* score is based on the Wilcoxon signed-rank test.

^i^ A group × time interaction effect was found; therefore, further analyses were performed (eFigure 3 in Supplement 1).

^j^ The *z* score is based on the Mann-Whitney test comparing 6-month values between improvers and nonimprovers. Data were available for 125 participants.

^k^ The *z* score is based on the Mann-Whitney test comparing 6-month values between improvers and nonimprovers. Data were available for 124 participants.

^l^ Complete data on both time points were available for 106 participants.

Furthermore, a group × time interaction effect for adiponectin (*F*_1,98_ = 9.49; *P* = .003), TNF (*F*_1,108_ = 5.97; *P* = .02), and IL-6 (*F*_1,108_ = 7.27; *P* = .01) was shown. Only cognitive improvers showed higher median adiponectin levels after bariatric surgery (improvers, from 2.3 μg/mL [IQR, 1.7-2.9 μg/mL] to 2.6 μg/mL [IQR, 1.9-3.7 μg/mL]; *P* < .001; nonimprovers, from 2.4 μg/mL [IQR, 1.9-2.7 μg/mL] to 2.4 μg/mL [IQR, 2.1-2.9 μg/mL]; *P* = .38) (Table 3; eFigure 3 in Supplement 1). However, no significant differences were observed between improvers and nonimprovers at both time points in adiponectin level (baseline, *P* = .11; at 6 months, *P* = .14). Only nonimprovers showed a significant change in median TNF and IL-6 levels over time (TNF: improvers, from 3.9 pg/mL [IQR, 3.0-5.2 pg/mL] to 3.8 pg/mL [IQR, 3.3-4.5 pg/mL]; *P* = .39; nonimprovers, from 4.4 pg/mL [IQR, 3.4-5.7 pg/mL] to 3.2 pg/mL [IQR, 2.7-4.2 pg/mL]; *P* < .001; and IL-6: improvers, from 1.7 pg/mL [IQR, 1.3-2.8 pg/mL] to 1.6 pg/mL [IQR, 1.2-2.4 pg/mL]; *P* = .16; nonimprovers, from 2.5 pg/mL [IQR, 1.4-3.6 pg/mL] to 1.6 pg/mL [IQR, 0.9-2.1 pg/mL]; *P* < .001) (Table 3; eFigure 3 in Supplement 1). Anthropometric measurements and physical activity did not differ between both groups. eTable 4 in Supplement 1 shows the correlation coefficients between changes in cognitive test scores and changes in obesity indices, plasma markers, BDI, and physical activity. Change in BMI and WC were both positively correlated with change in verbal fluency (BMI: *r* = 0.21; *P* = .02; WC: *r* = 0.23; *P* = .02). Change in TNF level was positively correlated with improvement in working memory (*r* = 0.18; *P* = .04), and change in IL-6 level was positively correlated with episodic memory (*r* = 0.18; *P* = .049).

## Discussion

**Figure 2.**
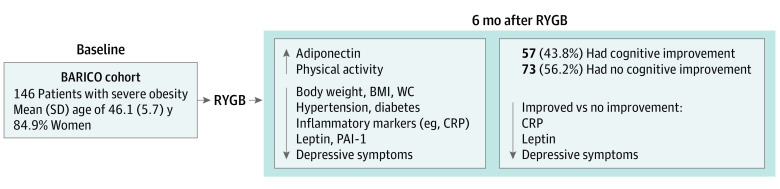
Schematic Summary of the Main Results BARICO indicates Bariatric Surgery Rijnstate and Radboudumc Neuroimaging and Cognition in Obesity; BMI, body mass index; CRP, C-reactive protein; PAI-1, plasminogen activator inhibitor 1; RYGB, Roux-en-Y gastric bypass; and WC, waist circumference.

In this study, we wanted to reveal the underlying processes involved in cognitive improvement after bariatric surgery. Cognitive improvement was observed for all cognitive domains after surgery. Taking repeated testing into account, 43.8% of the participants showed improvement in overall cognitive function. Furthermore, 6 months after bariatric surgery, significant improvements in general health were observed, such as less inflammation; lowered leptin, SAA, and PAI-1 levels; higher adiponectin level; lower blood pressure; less use of medication for comorbidities (such as diabetes and hypertension); less reported use of antidepressants; improved mood; and higher physical activity levels (Figure 2). Participants classified as cognitive improvers had lower CRP and leptin levels and fewer depressive symptoms after surgery than nonimprovers. This association was independent of physical activity and anthropometric measures.

Consistent with past studies, our results indicate an association between cognitive improvement and bariatric surgery.^5,7,8^ Most earlier studies did not control for repeated testing. In this study, we used the 20% change index, which demonstrated that only part of the participants showed reliable cognitive improvement according to this relatively strict definition, although the reliable change index or the inclusion of a control group remains the preferred method to study cognitive changes. Studies that were performed earlier often described improvement in memory, executive function, and cognitive control after bariatric surgery.^5,6^ The largest effect sizes in our study were found for the cognitive domains of verbal fluency and ability to shift attention (ie, executive functions). Larger changes in BMI and WC were associated with a higher change in the ability to shift attention as well. This finding is supported by a meta-analysis showing deficits in executive functions associated with obesity.^35^ Our results suggest that obesity-associated cognitive decline is at least partly reversible by bariatric surgery. Based on the 20% change index, we found reliable cognitive improvement in approximately 39% of the participants. These participants had lower baseline cognitive test scores, indicating either a higher range of improvement in these lower-scoring individuals or a higher test sensitivity toward change in this group. They also showed improved liver (CRP) and white adipose tissue–related (leptin) biomarkers, confirming large heterogeneity in obesity.^36^

Lower CRP and leptin levels and fewer depressive symptoms were observed in participants with cognitive improvement compared with nonimprovers, although nonimprovers did show lower CRP and leptin levels and fewer depressive symptoms as well. CRP is a liver-specific and highly sensitive inflammation marker. Results regarding CRP with higher leptin levels suggest that concerted beneficial associations with biomarkers of the liver and white adipose tissue are seen among cognitive improvers. This finding indicates the importance of improvement in metabolic function for cognitive improvement. Furthermore, an interaction was observed for adiponectin, TNF, and IL-6. Cognitive improvers did not show less TNF and IL-6 over time, while nonimprovers did. However, TNF and IL-6 values were within the normal range for healthy adults for both groups, making these differences less relevant.^37,38^ Adiponectin levels were higher among improvers after bariatric surgery, but not among nonimprovers. Adiponectin is an important adipokine with multiple functions, such as anti-inflammatory effects, inhibiting formation of atherosclerosis, and glucose regulation, but it also shows neuroprotective effects.^15,39,40^ Adiponectin seems to be associated with memory performance.^41^ Altogether, our results indicate the importance of improvement in liver and white adipose tissue functioning for cognitive improvement.

One of the most pronounced outcomes of bariatric surgery that was also observed in our study is reduced obesity-related comorbidities, such as hypertension and diabetes. Remission of hypertension has a positive influence on vascular wall health and might be associated with improved blood circulation and cerebral blood flow.^42,43^ Cognitive functioning is generally correlated with cerebral blood flow,^44^ implying that cerebrovascular changes after bariatric surgery may be associated with cognitive improvement. Structural brain changes in obesity, such as reduced hippocampal and gray matter volume, have been linked to impaired glucose and insulin regulation,^45^ suggesting that improvement in diabetes and glucose regulation might also be associated with cognitive improvement after bariatric surgery. Whether cognitive improvement after bariatric surgery is associated with cerebrovascular function and glycemic control needs to be further investigated.

Earlier studies focused on separate associations of CRP and leptin levels with cognitive functioning after bariatric surgery. A study in a comparable patient population did not find an association between CRP level and cognitive improvement after surgery; however, there were some differences compared with our study^19^ (ie, differences in age range of patients and statistical analysis), and they tested multiple domains separately instead of using 1 global cognitive score.^19^ These differences and the fact that we divided the participants into 2 groups based on overall cognitive performance might explain the discrepancy in results. Other studies support the hypothesis that lower cognitive function among individuals with obesity is associated with systemic inflammation.^17,18,46^ Our results regarding leptin are consistent with an earlier study in which lower leptin levels 12 months after bariatric surgery were associated with better executive function.^47^

Fewer depressive symptoms were observed among cognitive improvers, indicating that cognitive functioning is associated with mood.^12^ The exact mechanism linking obesity, depression, and cognitive function is most likely complex because these factors are all interrelated, making causality difficult to establish.^4,12,48^ Physical activity did not differ between participants with and participants without cognitive improvement, ruling out physical activity as a potential factor underlying cognitive improvement. Overall, we did not find many differences between participants regarding change in physical activity level. The physical activity results might have been influenced by social desirability because self-reported data were used^49^; therefore, a more objective measurement for physical activity is advised in future studies.

### Limitations and Strengths

Our study has some limitations. First, due to a relatively short follow-up, it is not possible to suggest longer-term associations of weight loss with reductions in the risk of neurogenerative diseases. Second, beause this study lacks a control group, we had to control for possible practice or learning effects of repeated testing using standardized multiple versions of cognitive tests and the 20% change index. We believe that combining these 2 methods gives a good representation of actual cognitive improvement. Third, because this study is an observational study, it is not possible to draw conclusions on causality, and follow-up intervention studies are therefore planned. The study also has some strengths, including a large sample size, the dividing of the groups into cognitive improvers and nonimprovers, and the inclusion of multiple variables for better understanding of the factors associated with cognitive improvement after bariatric surgery.

## Conclusions

The results of this cohort study suggest an association between bariatric surgery and cognitive improvement for approximately 39% of the participants. Furthermore, lower inflammation, changes in adipokines, improved mood, and higher physical activity levels were seen. The observed changes in liver-specific inflammation (CRP levels), adipokines (leptin levels), and depressive symptoms might partly explain the mechanism behind cognitive improvement after bariatric surgery. The further reductions in CRP and leptin levels specifically among cognitive improvers indicate the importance of improving the metabolic-inflammatory condition of both organs to improve cognition. The exact interaction between individual biomarkers and how they are mechanistically associated with cognitive function in multiple domains remains unsolved. Future studies should include a control group and multiple other potential mechanisms to clarify cognitive improvement after bariatric surgery, such as cerebrovascular function, glycemic control, microbiota, eating disorders, or nutrition (eg, caloric restriction). Furthermore, longitudinal studies are needed to study cognition and weight loss over time and to investigate whether weight loss might reduce the risk of neurodegenerative diseases.

## Notes

Supplement 1. **eTable 1.** Baseline Characteristics and Cognitive Test Scores of Improvers and Non-improvers **eFigure 1.** Flow Chart of the Study **eTable 2.** Missing Data per Outcome Measure at Baseline and 6 Months After Bariatric Surgery **eTable 3.** Plasma Concentrations of Adipokines and Inflammatory Markers Before and 6 Months After Bariatric Surgery **eFigure 2.** Boxplots With Cognitive Outcomes Before and 6 Months After Bariatric Surgery (A-E) **eFigure 3.** Boxplots With Plasma Concentrations of Adipokines and Inflammatory Markers per Group (Improvers and Non-improvers) Before and 6 Months After Bariatric Surgery (A-C) **eTable 4.** Pearson and Spearman Correlation Coefficients Between Changes in Cognition and Changes in Anthropometric Measures, Plasma Levels, Mood and Physical Activity

Supplement 2.
Data Sharing Statement

## References

[1] World Health Organization. Obesity and overweight. Updated June 9, 2021. Accessed December 2021. https://www.who.int/news-room/fact-sheets/detail/obesity-and-overweight

[2] Smith E, Hay P, Campbell L, Trollor JN. A review of the association between obesity and cognitive function across the lifespan: implications for novel approaches to prevention and treatment. Obes Rev. 2011;12(9):740-755. doi:10.1111/j.1467-789X.2011.00920.x 21991597

[3] Fitzpatrick AL, Kuller LH, Lopez OL, . Midlife and late-life obesity and the risk of dementia: cardiovascular health study. Arch Neurol. 2009;66(3):336-342. doi:10.1001/archneurol.2008.582 19273752 PMC3513375

[4] Dye L, Boyle NB, Champ C, Lawton C. The relationship between obesity and cognitive health and decline. Proc Nutr Soc. 2017;76(4):443-454. doi:10.1017/S0029665117002014 28889822

[5] Handley JD, Williams DM, Caplin S, Stephens JW, Barry J. Changes in cognitive function following bariatric surgery: a systematic review. Obes Surg. 2016;26(10):2530-2537. doi:10.1007/s11695-016-2312-z 27468905

[6] Thiara G, Cigliobianco M, Muravsky A, . Evidence for neurocognitive improvement after bariatric surgery: a systematic review. Psychosomatics. 2017;58(3):217-227. doi:10.1016/j.psym.2017.02.004 28410777

[7] Alosco ML, Galioto R, Spitznagel MB, . Cognitive function after bariatric surgery: evidence for improvement 3 years after surgery. Am J Surg. 2014;207(6):870-876. doi:10.1016/j.amjsurg.2013.05.018 24119892 PMC3983172

[8] Gunstad J, Strain G, Devlin MJ, . Improved memory function 12 weeks after bariatric surgery. Surg Obes Relat Dis. 2011;7(4):465-472. doi:10.1016/j.soard.2010.09.015 21145295 PMC3117085

[9] Georgiadou E, Gruner-Labitzke K, Köhler H, de Zwaan M, Müller A. Cognitive function and nonfood-related impulsivity in post-bariatric surgery patients. Front Psychol. 2014;5:1502. doi:10.3389/fpsyg.2014.01502 25566164 PMC4271510

[10] Prehn K, Profitlich T, Rangus I, . Bariatric surgery and brain health—a longitudinal observational study investigating the effect of surgery on cognitive function and gray matter volume. Nutrients. 2020;12(1):127. doi:10.3390/nu12010127 31906475 PMC7019777

[11] Nota MHC, Vreeken D, Wiesmann M, Aarts EO, Hazebroek EJ, Kiliaan AJ. Obesity affects brain structure and function—rescue by bariatric surgery? Neurosci Biobehav Rev. 2020;108:646-657. doi:10.1016/j.neubiorev.2019.11.025 31794778

[12] Chepenik LG, Cornew LA, Farah MJ. The influence of sad mood on cognition. Emotion. 2007;7(4):802-811. doi:10.1037/1528-3542.7.4.802 18039049

[13] Hillman CH, Erickson KI, Kramer AF. Be smart, exercise your heart: exercise effects on brain and cognition. Nat Rev Neurosci. 2008;9(1):58-65. doi:10.1038/nrn2298 18094706

[14] Cypess AM. Reassessing human adipose tissue. N Engl J Med. 2022;386(8):768-779. doi:10.1056/NEJMra2032804 35196429

[15] Kiliaan AJ, Arnoldussen IA, Gustafson DR. Adipokines: a link between obesity and dementia? Lancet Neurol. 2014;13(9):913-923. doi:10.1016/S1474-4422(14)70085-7 25142458 PMC4228955

[16] Skurk T, Alberti-Huber C, Herder C, Hauner H. Relationship between adipocyte size and adipokine expression and secretion. J Clin Endocrinol Metab. 2007;92(3):1023-1033. doi:10.1210/jc.2006-1055 17164304

[17] Lasselin J, Magne E, Beau C, . Low-grade inflammation is a major contributor of impaired attentional set shifting in obese subjects. Brain Behav Immun. 2016;58:63-68. doi:10.1016/j.bbi.2016.05.013 27223095

[18] Sweat V, Starr V, Bruehl H, . C-reactive protein is linked to lower cognitive performance in overweight and obese women. Inflammation. 2008;31(3):198-207. doi:10.1007/s10753-008-9065-3 18347963 PMC3116730

[19] Hawkins MA, Alosco ML, Spitznagel MB, . The association between reduced inflammation and cognitive gains after bariatric surgery. Psychosom Med. 2015;77(6):688-696. doi:10.1097/PSY.0000000000000125 25478707 PMC4456339

[20] Tsai CL, Huang TH, Tsai MC. Neurocognitive performances of visuospatial attention and the correlations with metabolic and inflammatory biomarkers in adults with obesity. Exp Physiol. 2017;102(12):1683-1699. doi:10.1113/EP086624 28983981

[21] Vreeken D, Wiesmann M, Deden LN, . Study rationale and protocol of the BARICO study: a longitudinal, prospective, observational study to evaluate the effects of weight loss on brain function and structure after bariatric surgery. BMJ Open. 2019;9(1):e025464. doi:10.1136/bmjopen-2018-025464 30782752 PMC6340014

[22] World Medical Association. World Medical Association Declaration of Helsinki: ethical principles for medical research involving human subjects. JAMA. 2013;310(20):2191-2194. doi:10.1001/jama.2013.281053 24141714

[23] Brookwood Medical Publications. ICH Harmonised Tripartite Guideline for Good Clinical Practice. Brookwood Medical Publications Ltd; 1996.

[24] Landelijk Trial Register. The effects of weight loss after bariatric surgery on brain function and structure. Accessed April 21, 2023. https://www.clinicaltrialregister.nl/nl/trial/28949

[25] Nasreddine ZS, Phillips NA, Bédirian V, . The Montreal Cognitive Assessment, MoCA: a brief screening tool for mild cognitive impairment. J Am Geriatr Soc. 2005;53(4):695-699. doi:10.1111/j.1532-5415.2005.53221.x 15817019

[26] Wechsler D. Wechsler Adult Intelligence Scale. 4th ed (WAIS-IV). NCS Pearson; 2008:498.

[27] Wilson BA, Cockburn J, Baddeley AD. *The Rivermead Behavioural Memory Test*. Thames Valley Test Co; 1991.

[28] Schmand B, Groenink SC, van den Dungen M. Letterfluency: psychometrische eigenschappen en Nederlandse normen. Tijdschr Gerontol Geriatr. 2008;39(2):64-76. doi:10.1007/BF03078128 18500167

[29] Zimmermann P, Fimm B. TAP–Test of Attentional Performance. PsyTest. Accessed April 19, 2023. https://www.psytest.net/en/test-batteries/tap/subtests

[30] Collie A, Darby DG, Falleti MG, Silbert BS, Maruff P. Determining the extent of cognitive change after coronary surgery: a review of statistical procedures. Ann Thorac Surg. 2002;73(6):2005-2011. doi:10.1016/S0003-4975(01)03375-6 12078822

[31] Verhage F. Intelligentie en Leeftijd: Onderzoek bij Nederlanders van Twaalf tot Zevenenzeventig Jaar. Van Gorcum; 1964.

[32] International Standard Classification of Education: ISCED 2011. UNESCO Institute for Statistics. Accessed July 18, 2022. https://uis.unesco.org/sites/default/files/documents/international-standard-classification-of-education-isced-2011-en.pdf

[33] Beck AT, Ward CH, Mendelson M, Mock J, Erbaugh J. An inventory for measuring depression. Arch Gen Psychiatry. 1961;4:561-571. doi:10.1001/archpsyc.1961.01710120031004 13688369

[34] Baecke JA, Burema J, Frijters JE. A short questionnaire for the measurement of habitual physical activity in epidemiological studies. Am J Clin Nutr. 1982;36(5):936-942. doi:10.1093/ajcn/36.5.936 7137077

[35] Yang Y, Shields GS, Guo C, Liu Y. Executive function performance in obesity and overweight individuals: a meta-analysis and review. Neurosci Biobehav Rev. 2018;84:225-244. doi:10.1016/j.neubiorev.2017.11.020 29203421

[36] Brandão I, Martins MJ, Monteiro R. Metabolically healthy obesity—heterogeneity in definitions and unconventional factors. Metabolites. 2020;10(2):48. doi:10.3390/metabo10020048 32012784 PMC7074352

[37] Said EA, Al-Reesi I, Al-Shizawi N, . Defining IL-6 levels in healthy individuals: a meta-analysis. J Med Virol. 2021;93(6):3915-3924. doi:10.1002/jmv.26654 33155686

[38] Li Y, Yi JS, Russo MA, Rosa-Bray M, Weinhold KJ, Guptill JT. Normative dataset for plasma cytokines in healthy human adults. Data Brief. 2021;35:106857. doi:10.1016/j.dib.2021.106857 33665253 PMC7900339

[39] Wang ZV, Scherer PE. Adiponectin, cardiovascular function, and hypertension. Hypertension. 2008;51(1):8-14. doi:10.1161/HYPERTENSIONAHA.107.099424 17998473

[40] Rizzo MR, Fasano R, Paolisso G. Adiponectin and cognitive decline. Int J Mol Sci. 2020;21(6):2010. doi:10.3390/ijms21062010 32188008 PMC7139651

[41] Cezaretto A, Suemoto CK, Bensenor I, Lotufo PA, de Almeida-Pititto B, Ferreira SRG; ELSA Research Group. Association of adiponectin with cognitive function precedes overt diabetes in the Brazilian Longitudinal Study of Adult Health: ELSA. Diabetol Metab Syndr. 2018;10:54. doi:10.1186/s13098-018-0354-1 30002734 PMC6038247

[42] Tschoner A, Sturm W, Gelsinger C, . Long-term effects of weight loss after bariatric surgery on functional and structural markers of atherosclerosis. Obesity (Silver Spring). 2013;21(10):1960-1965. doi:10.1002/oby.20357 23512491

[43] Wilhelm SM, Young J, Kale-Pradhan PB. Effect of bariatric surgery on hypertension: a meta-analysis. Ann Pharmacother. 2014;48(6):674-682. doi:10.1177/1060028014529260 24662112

[44] Rusinek H, Ha J, Yau PL, . Cerebral perfusion in insulin resistance and type 2 diabetes. J Cereb Blood Flow Metab. 2015;35(1):95-102. doi:10.1038/jcbfm.2014.173 25315860 PMC4294398

[45] Makaronidis JM, Batterham RL. Obesity, body weight regulation and the brain: insights from fMRI. Br J Radiol. 2018;91(1089):20170910. doi:10.1259/bjr.20170910 29365284 PMC6223152

[46] Spyridaki EC, Simos P, Avgoustinaki PD, . The association between obesity and fluid intelligence impairment is mediated by chronic low-grade inflammation. Br J Nutr. 2014;112(10):1724-1734. doi:10.1017/S0007114514002207 25315424

[47] Alosco ML, Spitznagel MB, Strain G, . Improved serum leptin and ghrelin following bariatric surgery predict better postoperative cognitive function. J Clin Neurol. 2015;11(1):48-56. doi:10.3988/jcn.2015.11.1.48 25628737 PMC4302179

[48] Carey M, Small H, Yoong SL, Boyes A, Bisquera A, Sanson-Fisher R. Prevalence of comorbid depression and obesity in general practice: a cross-sectional survey. Br J Gen Pract. 2014;64(620):e122-e127. doi:10.3399/bjgp14X677482 24567650 PMC3933857

[49] Prince SA, Adamo KB, Hamel ME, Hardt J, Connor Gorber S, Tremblay M. A comparison of direct versus self-report measures for assessing physical activity in adults: a systematic review. Int J Behav Nutr Phys Act. 2008;5:56. doi:10.1186/1479-5868-5-56 18990237 PMC2588639

