# Supplementary material for: Factors Associated With Cognitive Improvement After Bariatric Surgery Among Patients With Severe Obesity in the Netherlands
Source: JAMA Netw Open. 2023 May 30;6(5):e2315936. doi: 10.1001/jamanetworkopen.2023.15936 (PMC10230316; doi:10.1001/jamanetworkopen.2023.15936)
Supplement: Supplement 1. — eTable 1. Baseline Characteristics and Cognitive Test Scores of Improvers and Non-improvers eFigure 1. Flow Chart of the Study eTable 2. Missing Data per Outcome Measure at Baseline and 6 Months After Bariatric Surgery eTable 3. Plasma Concentrations of Adipokines and Inflammatory Markers Before and 6 Months After Bariatric Surgery eFigure 2. Boxplots With Cognitive Outcomes Before and 6 Months After Bariatric Surgery (A-E) eFigure 3. Boxplots With Plasma Concentrations of Adipokines and Inflammatory Markers per Group (Improvers and Non-improvers) Before and 6 Months After Bariatric Surgery (A-C) eTable 4. Pearson and Spearman Correlation Coefficients Between Changes in Cognition and Changes in Anthropometric Measures, Plasma Levels, Mood and Physical Activity [file jamanetwopen-e2315936-s001.pdf]

Supplementary Online Content

Vreeken D, Seidel F, Custers EM, et al. Factors associated with cognitive improvement after bariatric surgery among patients with severe obesity in the Netherlands. *JAMA Netw Open.* 2023;6(5):e2315936. doi:10.1001/jamanetworkopen.2023.15936

eTable 1. Baseline Characteristics and Cognitive Test Scores of Improvers and Non-improvers
eFigure 1. Flow Chart of the Study
eTable 2. Missing Data per Outcome Measure at Baseline and 6 Months After Bariatric Surgery
eTable 3. Plasma Concentrations of Adipokines and Inflammatory Markers Before and 6 Months After Bariatric Surgery
eFigure 2. Boxplots With Cognitive Outcomes Before and 6 Months After Bariatric Surgery (A-E)
eFigure 3. Boxplots With Plasma Concentrations of Adipokines and Inflammatory Markers per Group (Improvers and Non-improvers) Before and 6 Months After Bariatric Surgery (A-C)
eTable 4. Pearson and Spearman Correlation Coefficients Between Changes in Cognition and Changes in Anthropometric Measures, Plasma Levels, Mood and Physical Activity

This supplementary material has been provided by the authors to give readers additional information about their work.

eTable 1. Baseline Characteristics and Cognitive Test Scores of Improvers and Non-improvers

	Improvers (n=57)	Non-improvers (n=73)	t/Chi-square	p-value
**Baseline characteristics**				
Age, mean ± SD (y)	45.4 ± 5.6	46.2 ± 5.8	0.72	0.47
Sex, women, n (%)	46 (80.7%)	64 (87.7%)	1.19	0.27
Level of education, n (%)				
Low	4	6	3.28	0.19
Middle	37	36		
High	16	31		
**Cognition, mean ± SD**				
**Digit Span (sum of Forward, Backward and Sorting)**	24.5 ± 4.3	27.4 ± 4.9	3.44	<0.001
**Story Recall (sum of immediate and delayed recall)**	14.9 ± 6.4	18.8 ± 5.3	4.28	<0.001
**COWAT**	34.1 ± 10.2	40.6 ± 10.1	3.62	<0.001
**TAP Flexibility index score**	-4.5 ± 8.3	-1.6 ± 8.0	2.02	<0.05
**Compound Z-score**	-0.29 ± 0.59	0.26 ± 0.64	5.09	<0.001

P-value of difference between improvers and non-improvers based on independent sample t-test or chi-square. Significant changes between groups are indicated by underscoring the F/Z values and corresponding p-values. Abbreviations: COWAT = Controlled Oral Word Association Test, TAP = Tests of Attentional Performance.

© 2023 Vreeken D et al. *JAMA Network Open.*

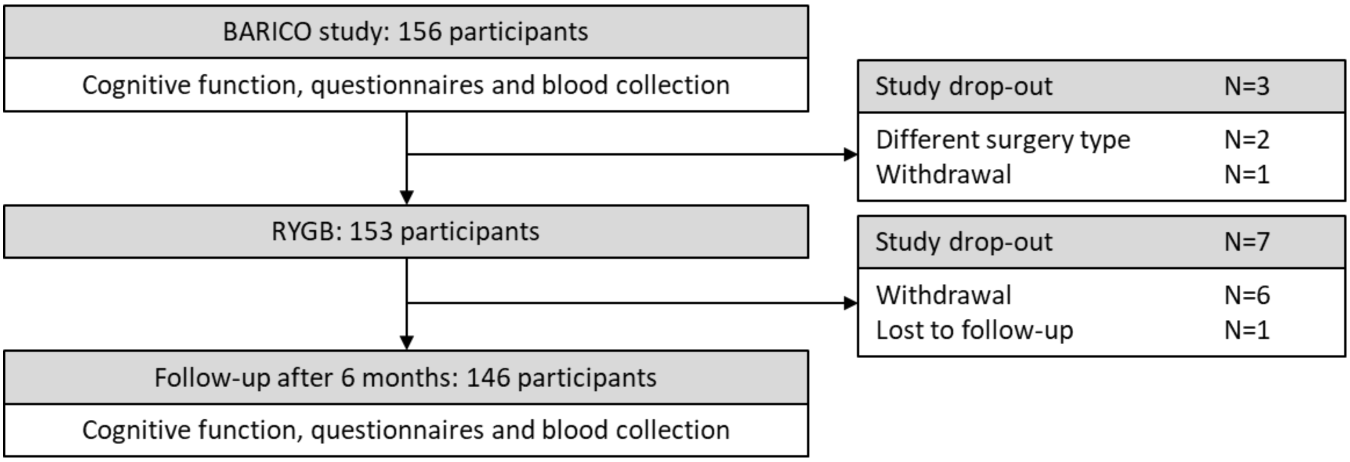

eFigure 1. Flow Chart of the Study**. 156 participants were recruited and underwent baseline measurements**.
In total, 146 participants underwent baseline and 6 months follow-up measurements including neuropsychological testing, questionnaires and blood collection. Abbreviations: RYGB = Roux-en-Y gastric bypass. eTable 2 shows more information on the missings per outcome measure.

© 2023 Vreeken D et al. *JAMA Network Open.*

eTable 2. Missing Data per Outcome Measure at Baseline and 6 Months After Bariatric Surgery

Outcome measure	Number of missings (%)	
	Baseline	6 months
**WC**[a]	14 (9.6%)	25 (17.1%)
**Blood pressure**[a]	0 (0%)	31 (21.2%)
**Plasma markers**		
CRP	16 (11.0%)	5 (3.4%)
Leptin	16 (11.0%)	16 (11.0%)
Adiponectin	16 (11.0%)	15 (10.3%)
SAA	17 (11.6%)	5 (3.4%)
TNF-α	16 (11.0%)	5 (3.4%)
IL-β	17 (11.6%)	5 (3.4%)
IL-6	16 (11.0%)	5 (3.4%)
PAI-1	16 (11.0%)	5 (3.4%)
**TAP Flexibility index score**	7 (4.8%)	9 (6.2%)
**BDI**	4 (2.7%)	8 (5.5%)
**Baecke**[b]	13 (8.9%)	25 (17.1%)

Only parameters with missing data are shown in the table. All other parameters did not contain any missing data on one of the two time points. [a] We had a lot of missing data for WC and blood pressure, as during the corona pandemic WC and blood pressure were not standard care anymore due to social distance. [b] Many patients did not fill in the complete questionnaire and therefore these data were not reliable and these scores were excluded. Abbreviations: WC = waist circumference, CRP = C-reactive protein, SAA = serum amyloid A, TNF-α = tumor necrosis factor alpha, IL-1β = interleukin-1β, IL-6 = interleukin-6, PAI-1 = plasminogen activator inhibitor 1, TAP = Tests of Attentional Performance, BDI-II = Beck Depression Inventory-Second Edition.

© 2023 Vreeken D et al. *JAMA Network Open.*

eTable 3. Plasma Concentrations of Adipokines and Inflammatory Markers Before and 6 Months After Bariatric Surgery

Plasma levels	Baseline (median (IQR))	6 months (median (IQR))	n	F/Z value	p-value
CRP (µg/ml)	4.8 (2.8 – 9.3)	1.4 (0.6 – 6.2)	125	360.62	<0.001
Leptin (pg/ml)	65.4 (51.3 – 85.7)	13.4 (8.5 - 22.9)	115	1230.03	<0.001
Adiponectin (µg/ml)	2.3 (1.7 – 2.8)	2.4 (1.9 – 3.1)	115	16.62	<0.001
SAA (µg/ml)	7.1 (5.1 – 16.5)	2.1 (1.1 – 4.0)	124	363.19	<0.001
TNF-α (pg/ml)	3.9 (3.0 – 5.3)	3.5 (2.8 – 4.2)	125	13.29	<0.001
IL-1β (pg/ml)	0.18 (0.07 – 0.36)	0.06 (0.00 – 0.20)	124	-4.62	<0.001
IL-6 (pg/ml)	2.1 (1.4 – 3.1)	1.5 (1.0 – 2.1)	125	24.05	<0.001
PAI-1 (ng/ml)	40.1 (29.8 – 57.2)	29.6 (21.1 – 44.1)	125	19.10	<0.001

Repeated measures analyses of variance were conducted to examine changes of time. The difference over time for IL-1β is based on the Wilcoxon Signed Rank Test.
Abbreviations: CRP = C-reactive protein, SAA = serum amyloid A, TNF-α = tumor necrosis factor alpha, IL-1β = interleukin-1β, IL-6 = interleukin-6, PAI-1 = plasminogen activator inhibitor 1.

© 2023 Vreeken D et al. *JAMA Network Open.*

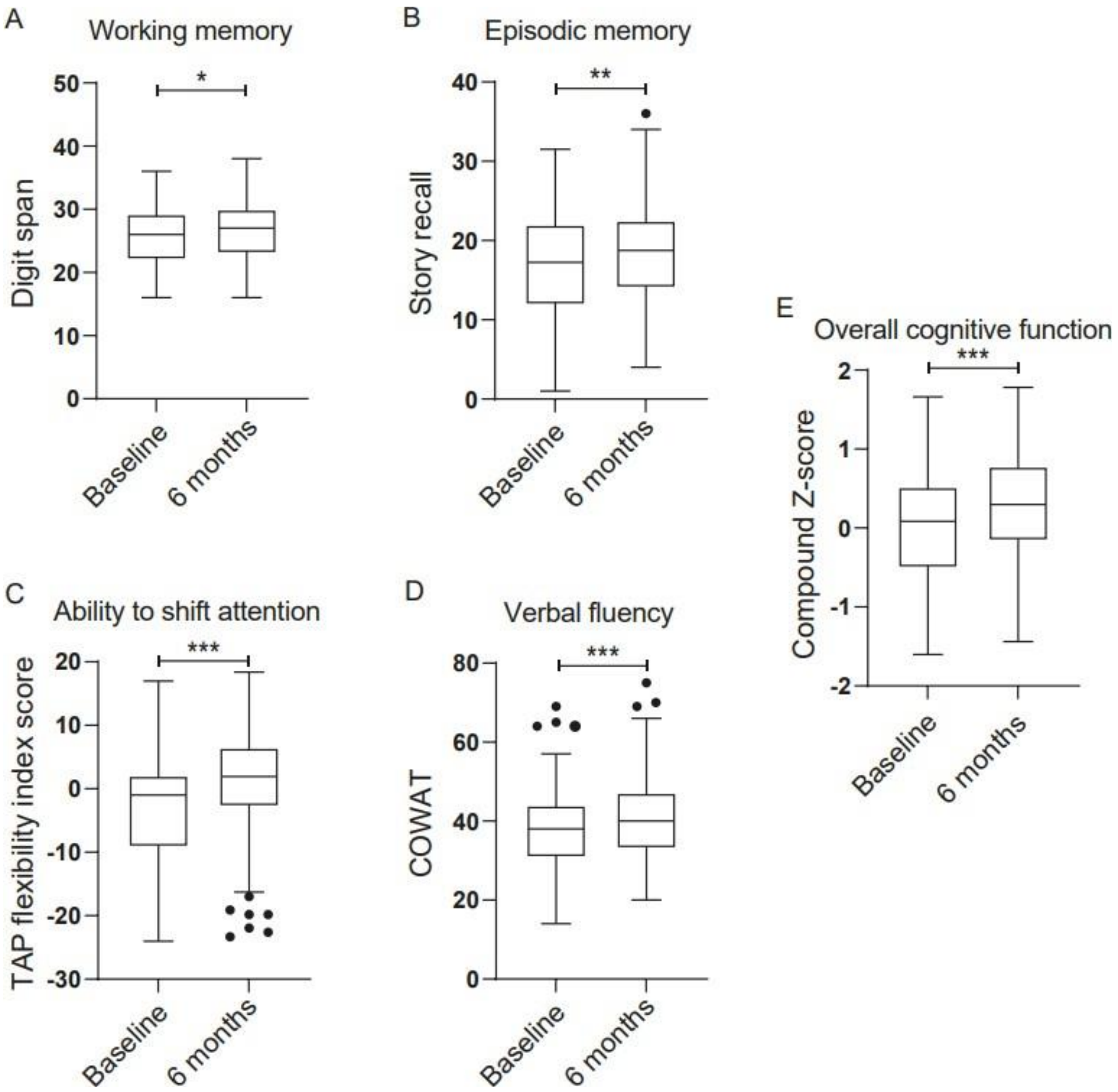

eFigure 2. Boxplots With Cognitive Outcomes Before and 6 Months After Bariatric Surgery (A-E)

Repeated measures analyses of variance were conducted to examine changes of time. *p<0.05, **p<0.01, ***p<0.001.
Data on both timepoints for the TAP flexibility index score (C) and Compound Z-score (E) were available for 130 participants.
Abbreviations: COWAT = Controlled Oral Word Association Test, TAP = Tests of Attentional Performance.

© 2023 Vreeken D et al. *JAMA Network Open.*

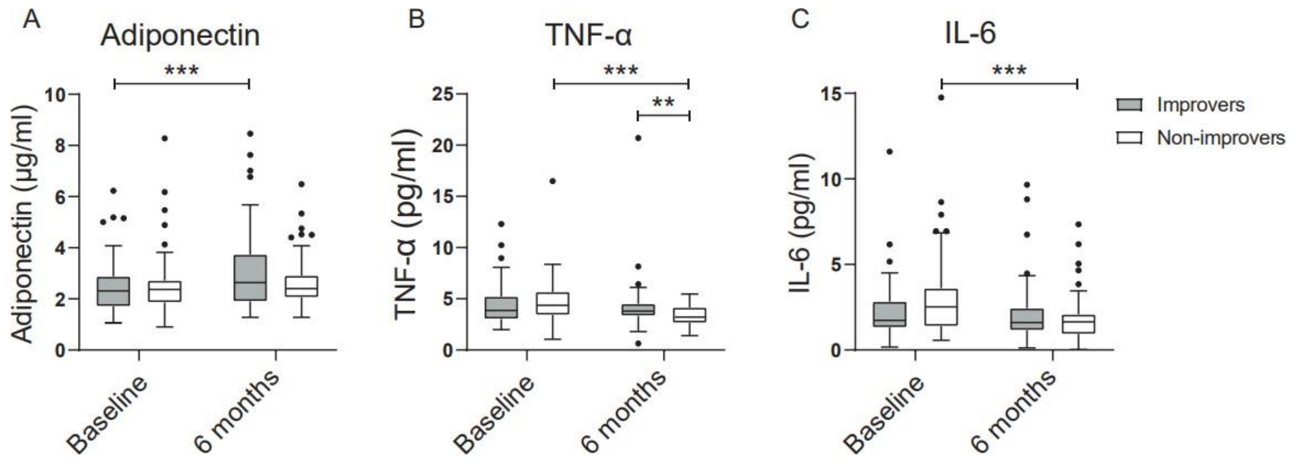

eFigure 3. Boxplots With Plasma Concentrations of Adipokines and Inflammatory Markers per Group (Improvers and Non-improvers) Before and 6 Months After Bariatric Surgery (A-C)

Repeated measures analyses of variance were conducted to examine changes of time and one-way analyses of variance were performed per timepoint to explore differences between improvers and non-improvers. **p<0.01, ***p<0.001.
Abbreviations: TNF-α = tumor necrosis factor alpha, IL-6 = interleukin-6.

© 2023 Vreeken D et al. *JAMA Network Open.*

eTable 4. Pearson and Spearman Correlation Coefficients Between Changes in Cognition and Changes in Anthropometric Measures, Plasma Levels, Mood and Physical Activity

	Delta Cognition				
	Digit Span (sum of Forward, Backward and Sorting)	Story Recall (sum of immediate and delayed recall)	COWAT	TAP Flexibility index score	Compound Z-score
**Delta Anthropometric measurements**					
**BMI (kg/m^2)**	0.01	-0.04	-0.11	0.21	0.06
**WC (cm)**	0.16	-0.02	-0.04	0.23	0.19
**Delta Blood pressure**					
**Systolic (mm HG)**	-0.17	-0.14	0.02	-0.12	-0.15
**Diastolic (mm HG)**	-0.11	-0.05	0.07	-0.09	-0.07
**Delta Plasma levels**					
**CRP (µg/ml)**	0.12	0.02	0.07	0.11	0.16
**Leptin (pg/ml)**	0.05	0.05	0.17	0.09	0.11
**Adiponectin (µg/ml)**	0.17	0.09	0.10	0.01	0.17
**SAA (µg/ml)**	0.06	-0.03	0.15	0.04	0.16
**TNF-α (pg/ml)**	0.18	-0.02	0.00	0.13	0.16
**IL-1β (pg/ml)**	0.02	-0.10	0.15	0.04	0.04
**IL-6 (pg/ml)**	0.06	0.18	0.03	-0.08	0.12
**PAI-1 (ng/ml)**	-0.08	-0.06	-0.05	0.18	0.06
**Delta BDI**	0.01	-0.08	-0.13	0.01	-0.11
**Delta Baecke**	0.06	0.12	-0.07	0.00	0.05

Pearson correlation coefficients and spearman correlation coefficients for CRP, SAA an IL-1β. Significant correlations are indicated by underscoring these correlation coefficients, $p<0.05$.
Abbreviations: COWAT = Controlled Oral Word Association Test, TAP = Tests of Attentional Performance, CRP = C-reactive protein, SAA = serum amyloid A, TNF-α = tumor necrosis factor alpha, IL-1β = interleukin-1β, IL-6 = interleukin-6, PAI-1 = plasminogen activator inhibitor 1, BDI = Beck Depression Inventory.

© 2023 Vreeken D et al. *JAMA Network Open.*
