# Supplementary material for: Factors Associated With Cognitive Improvement After Bariatric Surgery Among Patients With Severe Obesity in the Netherlands
Source: JAMA Netw Open. 2023 May 30;6(5):e2315936. doi: 10.1001/jamanetworkopen.2023.15936 (PMC10230316; doi:10.1001/jamanetworkopen.2023.15936)
Supplement: Supplement 2. — Data Sharing Statement [file jamanetwopen-e2315936-s002.pdf]

Data Sharing Statement

Vreeken. Factors Associated With Cognitive Improvement After Bariatric Surgery Among Patients With Severe Obesity in the Netherlands. *JAMA Netw Open*. Published May 30, 2023. doi:10.1001/jamanetworkopen.2023.15936

Data
Data available: No

Additional Information
Explanation for why data not available: De-identified data from the BARICO study will be made available upon request after approval by the study investigators.
